# The cell nucleus. A study in Burgundy

**DOI:** 10.1080/19491034.2019.1649835

**Published:** 2019-08-12

**Authors:** Oleg Demidov, Vasilisa Aksenova, Mary Dasso, Alexei Arnaoutov

**Affiliations:** a INSERM UMR1231, Laboratory of Excellence LipSTIC, University of Burgundy Franche-Comté, Dijon, France; b Insitutute of Cytology, Russian Academy of Sciences and Petrov Institute of Oncology, Saint-Petersburg, Russia; c Division of Molecular and Cellular Biology, Eunice Kennedy Shriver National Institute of Child Health and Human Development, NIH, Bethesda, MD, USA

**Keywords:** Phase separation, nuclear pores, nucleolus, lamins

## Abstract

Wilhelm Bernhard’s revolutionary microscopy techniques helped him put forward the hypothesis of specialized compartmentalization of the nucleus. He also described for the first time the nuclear bodies and peri-chromatin fibrils, and demonstrated that these granules contain an RNA component. The tradition of biennial workshops, named after this great scientist, continues, and this year it took place in the heart of Burgundy, in Dijon, France (May 20–24, 2019, organized by INSERM UMR1231, UBFC), where well-fed participants emphasized the importance of viewing the cell nucleus as a hub of specialized colloidal compartments that orchestrate replication, transcription and nuclear transport.

## Intracellular colloids

Ronald Hancock (Silesian Technical University, Poland) was awarded this year’s Wilhelm Bernhard Medal. During his lecture, he reminded the audience that biochemical reconstruction of endogenous reactions might be problematic if the effect of molecular crowding is not taken into consideration. We know that *in vivo* the concentrations of many macromolecules exceeds 100 mg/ml, a concentration at which the entropic forces induce greater interactions than occur in more dilute *in vitro* studies. Such interactions allow membrane-free colloid- or droplet-like structures to form from homo- or heterotypic complexes within both the cytoplasm and the nucleus. The core of a particular colloid would typically consist of an intrinsically disordered, low-complexity polypeptide, linked to an enzymatic or structural domain. Examples of such colloids include but are not limited to microscopically recognizable structures like RNA granules, PML bodies, and the nucleolus. Dr. Hancock’s survey of these principles and his contributions to them were warmly received.

In a similar vein, Karsten Weis (Institute of Biochemistry, Switzerland) described the fascinating journey of mRNA within or between processing bodies (PBs, stress granules) that form as colloid droplets assembled from proteins such as RNA helicase Dhh1 and adaptor proteins Pat1 and Lsm1. He further reported a tug-of-war between Pat1 and Not1 that controls PB dynamics: while Pat1 is essential for Dhh1-induced PB formation, it is Not1 that activates Dhh1 ATPase activity simultaneously disrupting these structures and releasing mRNA to associate with another enzyme(s) [1]. Strikingly, other RNA helicases, including Dbp1 and Ded1 are also capable of forming colloids in vitro. It will be interesting to determine whether an individual PB body is not a homogenous structure but rather represents an assembly of several individual colloid droplets, made of different adaptor-helicase complexes. If that were the case, then the directionality of mRNA translocation within or between PBs could be determined by the change of affinity of mRNA to a particular helicase, depending on the activity of the latter.

The importance of nuclear colloid droplets was underscored in the talks presented by Hugues de Thé and Valerie Lallemand-Breitenbach (College de France, INSERM-CNRS, France). Using their favorite model, PML (promyelocytic leukaemia) bodies, they demonstrated how cells may use colloids for efficient storage and retrieval of vital information. PML bodies are formed as hollow shells by the aggregation of PML protein, followed by its extensive SUMOylation, a post-translational modification that provides a platform for binding of a plethora of other proteins that possess SUMO-interacting motifs (SIMs). These SIM-containing clients fill the space within the PML sphere and, because many of them are SUMO substrates as well, form an intricate web of interactions with themselves and PML. Because SUMO de-modification is controlled by SUMO isopeptidases, this allows establishment of a dynamic hub, where a particular SIM-SUMO client protein accumulates and leaves the PML body, depending on its SUMOylation status [2]. Because many of PML clients are directly involved in various DNA damage repair processes, including p53, DAXX and SMC5/6, it comes as no surprise that PML bodies are viewed as potential targets for chemotherapy. Stunningly, simple chemicals – retinoic acid and arsenic – because of their ability to regulate PML stability, have emerged as powerful tools to treat and reverse PML-related diseases, like acute promyelocytic leukemia [3]. Dr. de Thé emphasized that these PML drugs could be used in combination with other therapeutics to battle other malignancies.

The current view of many intracellular structures as colloidal droplets emerged from the fact that all of them, although being spherical in nature, lack membrane components. It is therefore somewhat unexpected that some of these multimolecular condensates do indeed contain lipids. Two talks from Pavel Hozak’s group (Martin Sztacho and Sara Escudeiro-Lopes, Institute of Molecular Genetics, Czech Republic) described PIP2 (phosphatidylinositol 4,5-bisphosphate)-containing structures that are associated with intranuclear machineries, including the Pol II supercomplex and lamin A. Although determination of physiological significance of such interactions could be difficult, the presenters proposed that these newly-recognized PIP2 lipid islets are important for regulating the spatial partitioning of transcription and splicing of mRNAs [4].

## Nuclear pores and their function

The nuclear pore complexes (NPCs), that are also sometimes viewed as colloids, mediate the exchange of macromolecules between the nucleus and cytoplasm, and are composed of at least 30 different subunits, called nucleoporins. Though several nucleoporins within NPC are known to be highly dynamic, the whole structure was long considered to be quite rigid, and isolated NPCs from various organisms demonstrate remarkable similarities. Mary Dasso (NIH, USA) presented a new approach to study the function of individual subunits of a multi-protein complex. Her group utilizes the power of auxin-inducible degrons and CRISPR/Cas9 to endogenously tag and rapidly degrade individual nucleoporins. That combination allowed them to demonstrate that the whole, pre-formed NPC is mostly tolerant to the loss of its individual members, and that there are only a handful of linchpins that hold the complex together. These unanticipated findings allow the NPC to be considered not as an ensemble but rather as a small core of the linchpin components to which other nucleoporins may bind freely. This study also points to the possibility that even individual NPCs within a single cell might not be all the same and display some sort of heterogeneity.

Interestingly, the composition of NPCs is not static and its individual subunits may demonstrate dramatic rearrangements during an organism’s life. Yves Barral (Institute of Biochemistry, Switzerland) presented compelling evidence that age-associated changes in budding yeast are tightly linked to changes of NPC composition. His group showed that during asymmetric division, the mother cell retains accumulated by-products of DNA replication. For example, DNA repeat sequences may be excised from the genome and propagated as episomes, potentially posing a burden for replication machinery during the next cell cycle. The retention of these episomes in the mother cell is mediated by their specific tethering to the rearranged NPC, thus ensuring that the daughter cells do not receive them. The accumulation of episomes within the mother cell acts as an aging factor, eventually limiting the mother’s proliferative longevity.

The dynamism of the NPC constituents was also a central part of the talk presented by Shotaro Otsuka (Max F. Perutz Laboratories, Austria). He compared post-mitotic NPC assembly during telophase to NPC assembly within existing nuclear envelopes during interphase (i.e., without or with the pre-existing double nuclear membrane, respectively). Using endogenously tagged nucleoporins he demonstrated that these mechanisms are strikingly different: the initial steps of both processes may rely on transmembrane nucleoporin POM121 and the 10-subunit Y-subcomplex scaffold of the NPC, the timing and order of subsequent recruitment of other nucleoporins differs dramatically between the two events [5].

Consistent with the idea of the NPC as a hub of dynamic and multifunctional proteins, two talks highlighted the function of individual nucleoporins: Jana Uhlířová (Institute of Molecular Genetics, Czech Republic) presented evidence that Tpr, a nucleoporin associated with the nucleoplasmic face of the NPC, plays an essential role during cell specialization. In particular, myoblasts that lack this nucleoporin display profound defects during their differentiation into myotubes, indicating a possible function of Tpr as a transcriptional regulator. Irene Chiolo’s lab (University of South California, USA) continues the quest to define the mechanisms that are involved during repair of heterochromatin. Her talk showed that the re-localization of double-strand breaks in the heterochromatin to the repair sites at the nuclear periphery requires formation of special ‘rails’ that are formed by polymerized nuclear actin in response to the damage [6]. Intriguingly, the set of proteins required for this process includes several nucleoporins that dynamically interact with damaged DNA in the nucleoplasm and help recruiting and tethering it to the vicinity of the nuclear pores and lamins, where the repair takes place.

Tpr also appears to be involved in organization of lamina-associated heterochromatin domains (LAD), as depletion of Tpr results in disappearance of heterochromatin-exclusion zones from the nuclear pores [7]. Lamins play important, yet insufficiently understood roles in gene expression, although it is widely assumed that LADs are generally repressive [8]. Kseniya Perepelina (Almazov Medical Research Center, Russia) argued that LADs may not be universally repressive. She examined the effect of either wild type lamin A or a R527C mutant on induced osteogenic differentiation of four different cell lines, and found that the R527C mutant promoted different responses in the transcription program of different cells. Interestingly, the cell type-specific effect of lamins was also demonstrated by Igor Sharakhov (Virginia Polytechnic Institute, USA). Using *Drosophila* mutants in lamin B he showed that disruption of lamin-chromatin interactions resulted in cell type-specific effects on the spatial organization of chromatin.

## Single-molecule visualization

The emergence of technologies such as CRISPR/Cas9 and super-resolution microscopy allows analysis of both the localization and dynamics of DNA and proteins in living cells with unprecedented spatio-temporal resolution. Thoru Pederson (University of Massachusetts, USA) presented his group’s visualization of the dynamics of genomic loci that are situated in close proximity to each other on a single chromosome, kilobases apart. He described how this technology has revealed ongoing compaction-relaxation of chromosomal regions that coincides with the progression of the cell cycle [9].

Jurek Dobrucki (Jagiellonian University, Poland) reported live analysis of single-strand break (SSB) repair mechanisms. He proposed that the DNA repair protein XRCC1 forms PML-like foci in close proximity to the replication-associated SSBs. These foci contain other proteins involved in DNA damage repair, including PARP1 and 53BP1, possibly providing essential factors for the repair process [10]. The importance of colloid-like foci for DNA repair was also highlighted in the work presented by Olga Lavrik (Novosibirsk State University, Russia), who showed that poly(ADP-ribose) polymerase (PARP) catalyzes these polymers (PAR) at double-stranded DNA breaks. PAR associates with the RNA binding proteins YB-1 and FUS, thus possibly creating foci that may help in recruiting other factors required for efficient DNA repair. Poly(ADP-ribose) glycohydrolase (PARG) counteracts the formation of PAR polymers and helps to dissolve the foci once the repair is completed [11].

## Nucleosomes

The nucleosome is a structure that naturally restricts DNA accessibility for its reading by enzymes. Therefore, the search for the mechanisms that allow local or global uncoupling of the DNA-histone interface is a central question of chromosome biology. Gabor Szabo (University of Debrecen, Hungary) reported a cytometric-based pipeline using GFP-labelled histones that enables the quantitative analysis of nucleosome stability *in situ*, and he quantified the effects of histone variants, cell cycle and post-translational modifications on nucleosome stability using this novel method [12]. Alexey Onufriev (Virginia Commonwealth Technical University, USA) reported mathematical modeling of charge distributions within the DNA-histone interaction surface in the context of a nucleosome [13]. His group has built a comprehensive physics-based framework that predicts the effect of neutralization of charge in a particular histone lysine (by means of acetylation) on the level of DNA accessibility. As expected, most of the theoretically affected lysine residues are found to be situated in the relatively unexplored globular histone core, rather than in its tails. Although whether modifications of those particular lysine residues exist in nature remains unanswered, this work provides a strong structural framework that will justify searching for them. Daisuke Takahashi (Masahiko Harata lab, Tohoku University, Japan) also discussed the suppression of DNA accessibility by nucleosomes, and showed that two variants of H2A, namely its Z1 and Z2 isoforms, have distinct abilities to suppress gene activation. Elimination of one or the other resulted in different gene expression responses, especially under oxidative stress.

## Advances in cancer

Accumulating evidence indicates that the well-known activation of germline genes in tumors can disrupt the nuclear physiology of cells or lead to chromosomal instability, which is characteristic of cancer cells. Alexander Strunnikov (Guangzhou Institutes, China) demonstrated that germline cohesin complexes are potentially among such factors. He reported on meiotic cohesin subunits (mei-cohesins), describing an epigenomic assessment of mei-cohesins REC8, RAD21L, STAG3, and SMC1beta by ChIP-seq in normal macaque testis. He further showed that forced expression of mei-cohesin complexes in both normal and transformed somatic cell lines results in erroneous cohesion function and chromosome aberrations during mitotic segregation, suggesting that the physiological function of somatic cohesins differs substantially from their meiotic counterparts.

Sui Huang (Northwestern University, USA) discussed an intriguing connection between cancer progression and an enigmatic intranuclear structure, called the perinucleolar compartment (PNC). PNCs are usually detected only in cancer cells, and they are enriched in non-coding RNAs and RNA binding proteins. Her group discovered a small molecule, termed Metarrestin, that inhibits Pol I-mediated rDNA transcription, possibly through binding to translation elongation factor eEF1A2, and prevents formation of PNCs. Suppression of Pol I activity partially recapitulates the effect of Metarrestin, although the drug does not induce genotoxic stress in cells. Remarkably, Metarrestin proved to be very effective *in vivo*, as it substantially blocked formation of metastasis in both xenograft and PDX tumor models, without displaying adverse effects in healthy animals [14].

The Wilhelm Bernhard Workshops emphasize young people at the beginning of their scientific career and provide an open and welcoming environment for communication between students and established scientists. Over many years, the Wilhelm Bernhard Workshops have afforded young scientists their first experiences of presenting their data at international level, with abundant opportunities to receive feedback on their work, meet new people and establish collaborations. This tradition continues, with more than 40 students and young researchers attending the conference. We expect to hear the continuations of these projects and new ones coming at future Wilhelm Bernhard Workshops, and to watch as these young scientists become leaders within the field of nuclear biology.

Additional excellent talks at this meeting covered topics such as chromosomal organization, nuclear amyloids and intracellular signaling cascades. We regret that we were unable to cover all of them because of space limitations, and sincerely apologize to colleagues whose work could therefore not be described here. The abstracts of all presentations (oral and poster) were published in Biopolymers & Cell [15]. The next Wilhelm Bernhard Workshop on the Cell Nucleus will be held in Lviv, Ukraine in the summer of 2021.
